# Tumeur juvénile de la granulosa de l'ovaire: à propos d'un cas

**DOI:** 10.11604/pamj.2015.21.114.6453

**Published:** 2015-06-10

**Authors:** Ahmed Azahouani, Mohamed Balahcen

**Affiliations:** 1 CHU Mohamed VI, Oujda, Maroc

**Keywords:** Tumeur de la granulosa, ovaire, enfant, granulosa tumor, ovary, child

## Abstract

Ovarian sex cord tumors are rare tumors that develop at the expense of non-germ cell ovarian. The pathogenesis of these tumors remains undetermined and several cellular and molecular alterations may be involved in the development of juvenile granulosa cell tumors. For two decades, the individualization of juvenile granulosa cell tumors has been a major advance in the treatment of these children's tumors. However, their natural history is reported in the literature and through a short series and reliable prognostic factors are to establish. We report the case of a girl of 8 years who presented with abdominal-pelvic mass gradually increasing size, to surgical exploration found a huge abdominal mass at the expense of the left ovary. Histology and objective immunolabeling a tumor of the juvenile form of granulosa.

## Introduction

Les tumeurs de la granulosa dites juvéniles sont des tumeurs malignes appartenant au groupe des tumeurs des cordons sexuels et du stroma, elles sont rares et représentent moins de 5% des tumeurs ovariennes de l'enfant et de l'adolescente, avec un maximum de fréquence se situant entre 0 et 10 ans. Elles ont la particularité d’être dans la majorité des cas sécrétantes et hyperoestrogéniques, et sont considérées comme des tumeurs à bas potentiel de malignité.

## Patient et observation

**Figure 1:**
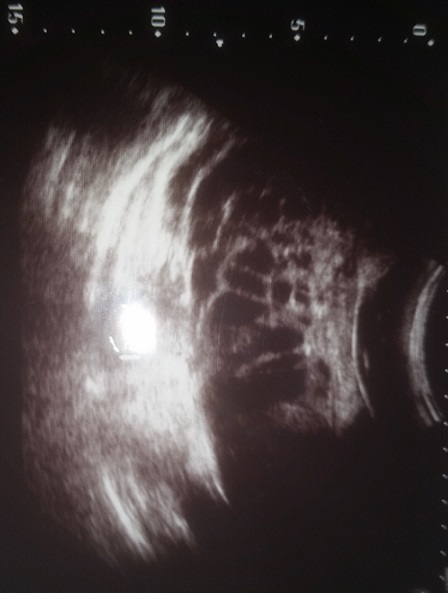
Aspect échographique d'une tumeur kystique multicloisonnée

Fille âgée de 8ans, sans antécédents pathologiques particuliers, elle accusait depuis environ 15 jours des douleurs pelviennes, paroxystiques, d'intensité modérée, associées à une augmentation du volume abdominal et un amaigrissement non chiffré. L'examen abdominal a trouvé une masse abdomino-pelvienne indolore mesurant approximativement 25 ×20 cm de diamètre, de consistance dure, mal limitée, mobile par rapport au plan superficiel, et fixe par rapport au plan profond, avec circulation veineuse collatérale, sans signes d'hypersécrétion et un ombilic non déplissé. L’échographie abdomino-pelvienne fut réalisée, qui a mis en évidence une volumineuse masse abdomino-pelvienne, occupant la quasi-totalité de l'abdomen, compatible avec une masse kystique supravésicale (Figure 1), associée à un épanchement intra-péritonéal de petite abondance, sans adénopathies profondes, et sans atteinte hépatique.

**Figure 2:**
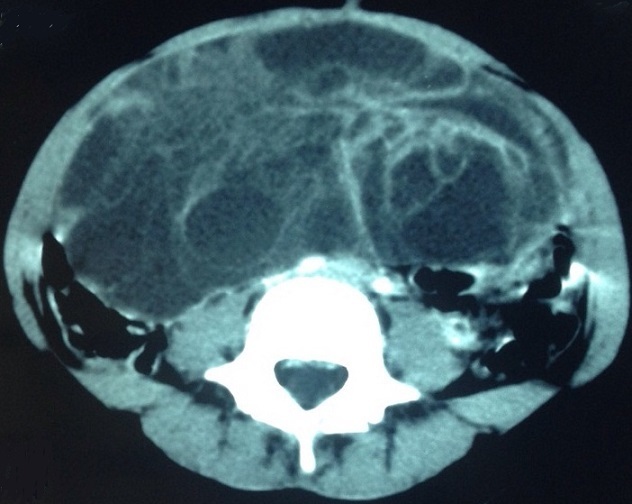
Coupe transversale de la tumeur de la granulosa

**Figure 3:**
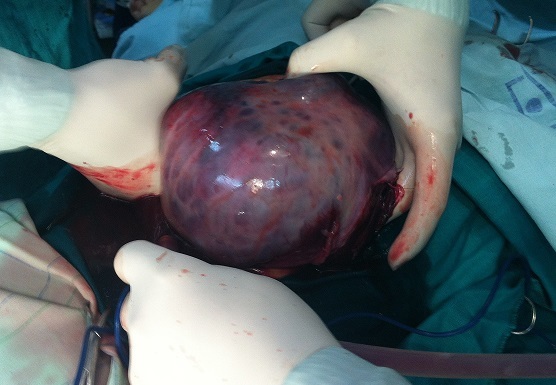
Tumeur en per-opératoire

**Figure 4:**
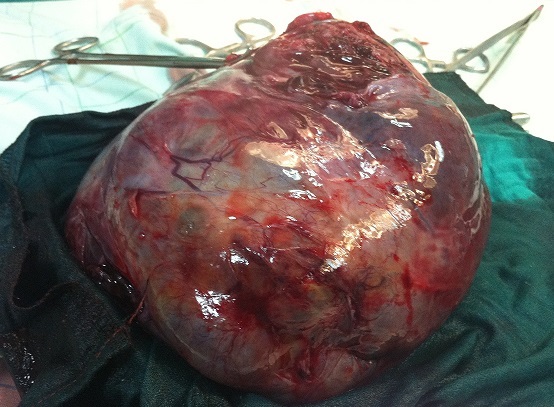
Masse d'exérèse

Une TDM abdomino-pelvienne a objectivé une masse kystique multicloisonnée, abdomino-pelvienne sus et latéro-vésicale gauche pouvant correspondre à un kyste ovarien gauche, une formation kystique d'autre nature ne peut être écartée (Figure 2). Les marqueurs tumoraux (Alpha-foetoprotéine et béta HCG) ont été normaux. L'exploration chirurgicale a trouvé une énorme tumeur au dépend de l'ovaire gauche de 25×20 cm (Figure 3, Figure 4), avec un épanchement séro-hématique. L’étude anatomo-pathologique complétée par l'immuno-marquage est revenue en faveur d'une tumeur juvénile de la granulosa.

Les suites opératoires étaient simples. La patiente a bénéficiée d'une surveillance périodique clinique et radiologique, par le biais de radiographies thoraciques et d’échographies abdomino-pelviennes. Ce suivi était sans particularités. Lors d'un staff multidisciplinaire on a décidé que la patiente ne nécessiterait pas de chimiothérapie ni de radiothérapie.

## Discussion

Les tumeurs de la granulosa juvéniles constituent une entité anatomopathologique particulière qui représente 5% des tumeurs ovariennes de l'enfant et de l'adolescente [1]. La forme juvénile des tumeurs de la granulosa est diagnostiquée chez des patientes de moins de 20ans dans 80% des cas, et moins de 10ans dans 50% des cas, d'où son appellation [2]. Plusieurs associations de tumeurs de la granulosa juvéniles avec des pathologies plus générales ont été rapportées; telles que l'endochondromatose d'Olliers, le syndrome de Maffucci, des anomalies dysplasiques [3]. Des altérations cellulaires et moléculaires sont impliquées dans le développement des TJG: rôle des facteurs de croissance et d'oncogènes, prolifération des cellules de la granulosa induite par la FSH et la Gαs, anomalie d'expression des gènes de la détermination gonadique [4]. Les tumeurs de la granulosa juvénile se manifestent par: un syndrome tumoral: distension abdominale douloureuse en rapport avec la taille de la tumeur [5]. Parfois, la douleur est aigue, résultant de la torsion de l'ovaire leure nature hémorragique. Exceptionnellement les tumeurs de la granulosa peuvent se présenter sous forme d'un tableau de rupture avec hémopéritoine et ceci du fait de leur nature hémorragique [1–5]; un syndrome endocrinien lié aux fonctions sécrétrices de ces tumeurs: pseudo-puberté précoce isosexuelle chez la jeune fille en cas de sécrétion oestrogénique; hirsutisme, hypertrophie clitoridienne en cas de sécrétion androgénique [6]. Dans le cas de notre patiente on a retrouvé la masse abdomino-pelvienne qui était isolée, sans signes d'hypersécrétion.

L’échographie est l'examen complémentaire le plus utilisé pour l'exploration des tumeurs ovariennes, elle permet de confirmer les données de l'examen clinique, de rattacher la masse pelvienne à son origine ovarienne, de déterminer ses caractères sémiologiques et d’évaluer le degré d'extension abdomino-pelvienne de la tumeur. L’échographie peut révéler une grande masse échogène, ou une masse kystique avec des cloisons, réalisant un aspect multiloculaire, mais l'aspect uniloculaire est retrouvé également, ou alors elle peut apparaître de nature solide pure homogène ou hétérogène [5].

La TDM a un taux de détection parfois inférieur de celui de l’échographie, dans le diagnostic de présomption des tumeurs ovariennes. Elle pourrait être justifiée devant une taille importante de la tumeur pelvienne qui pose le problème de son siège primitif et de ses rapports avec les structures anatomiques voisines [7]. Les renseignements fournis par l'IRM ne semblent pas supérieurs à ceux d'une échographie pelvienne réalisée dans d'excellentes conditions techniques par un échographiste expérimenté [7]. L’échographie et la TDM réalisées chez notre patiente ont précisées l'origine annexielle d'une énorme tumeur kystique multicloisonnée (Figure 1, Figure 2). L'estradiol est dosé en cas de pseudo-puberté précoce; il peut être utilisé comme marqueur tumoral, quant à l'inhibine, elle représente actuellement un bon marqueur spécifique des tumeurs de la granulosa [5].

La tumeur est en général unilatérale (97%) et mesure en moyenne 12.5 cm. Sa surface est lisse, sous-forme solide, kystique ou une association des deux formes. Elle est caractérisée par des plages denses de cellules à noyau non incisuré, hyperchromatique et souvent en mitose. De rares follicules immatures sécrétant du mucus sont observés. La lutéinisation est fréquente. Le marqueur à la vimentine est positif dans 80% des cas environ [1–8]. Ces aspects macroscopique et microscopique ont été retrouvés dans notre cas, avec une forte positivité de l'anticorps anti-Vimentine.

Le traitement idéal de ces tumeurs est chirurgical: l'annexectomie. La chimiothérapie est proposée en cas de récidive en complément d'une reprise chirurgicale, et doit utiliser au moins une anthracycline [9, 10]. La radiothérapie n'a pas fait la preuve de son efficacité [9, 10].

Dans notre cas la patiente a bénéficié d'une annexectomie gauche, sans complément de chimiothérapie (Figure 3, Figure 4). Les tumeurs de la granulosa juvénile sont généralement de très bon pronostic: 92% de survie à 5 ans [9, 10]. Les facteurs de mauvais pronostic sont: la taille importante, l'ascite et la rupture capsulaire [10].

## Conclusion

Les tumeurs de la granulosa sont des tumeurs rares de l'ovaire dont le diagnostic est anatomo-pathologique. Plusieurs altérations cellulaires et moléculaires pourraient être impliquées dans le développement de ces tumeurs. Le traitement comporte classiquement une chirurgie initiale, qui consiste en une ovariectomie ou salpingo-ovariectomie et le pronostic est généralement favorable.
